# Eight immune-related genes predict survival outcomes and immune characteristics in breast cancer

**DOI:** 10.18632/aging.103753

**Published:** 2020-08-03

**Authors:** Han Xu, Gangjian Wang, Lili Zhu, Hong Liu, Bingjie Li

**Affiliations:** 1 The Department of Breast Surgery, The First Affiliated Hospital of Zhengzhou University, Zhengzhou, Henan, China; 2 The Department of Oncology, The First Affiliated Hospital of Zhengzhou University, Zhengzhou, Henan, China

**Keywords:** breast cancer, immune checkpoint, immune cell, risk score, TCGA

## Abstract

Advancements in immunotherapy have improved our understanding of the immune characteristics of breast cancer. Here, we analyzed gene expression profiles and clinical data obtained from The Cancer Genome Atlas database to identify genes that were differentially expressed between breast tumor tissues and normal breast tissues. Comparisons with the Immunology Database and Analysis Portal (ImmPort) indicated that many of the identified differentially expressed genes were immune-related. Risk scores calculated based on an eight-gene signature constructed from these immune-related genes predicted both overall survival and relapse-free survival outcomes in breast cancer patients. The predictive value of the eight-gene signature was validated in different breast cancer subtypes using external datasets. Associations between risk score and breast cancer immune characteristics were also identified; *in* *vitro* experiments using breast cancer cell lines confirmed those associations. Thus, the novel eight-gene signature described here accurately predicts breast cancer survival outcomes as well as immune checkpoint expression and immune cell infiltration processes.

## INTRODUCTION

Breast cancer is a life-threatening disease of increasing clinical concern worldwide [1]. It is classified into four molecular subtypes; luminal A, luminal B, triple-negative breast cancer (TNBC), and human epidermal growth factor receptor type 2 (HER2) positive [2, 3]. Treatment methods are very different for each subtype [4]. Endocrine therapy is effective for treating luminal A and B subtypes, which are therefore associated with good prognoses. In addition, HER-2-positive breast cancer is sensitive to chemotherapy and anti-HER-2 therapy. However, prognoses are poor for TNBC because neither endocrine nor anti-HER-2 therapies are effective in this subtype. Thus, effective treatments for TNBC are urgently needed [5]. TNBC is characterized by genomic instability, high mutation load, and high levels of immune infiltration. Some clinical studies have therefore examined immunotherapy treatments for TNBC in recent years.

Immunotherapies can target both innate and adaptive immune mechanisms in the treatment of breast cancer [5, 6]. Immunotherapy techniques, especially those targeting PD1 and PDL-1, have been considered for use in clinical practice [7]. Although immunotherapy is a promising treatment method for breast cancer, many issues still need to be addressed. Immune evasion is a key problem in breast cancer immunotherapy, and it is further complicated by substantial differences in immune cell infiltration processes and immune response in breast cancer compared to other types of cancer [8, 9]. Additional research is needed to identify immune checkpoints and immune cell infiltration processes that could serve as treatment targets.

Some previously identified immune-related genes and cells with prognostic value in breast cancer patients might be effective immunotherapy targets. For example, the immune-related gene TGFBR2 predicts prognosis in estrogen receptor-negative patients after chemotherapy. Several other genes identified as potential targets for cancer treatment play important roles in immune responses [10–12]. In addition, colocalization of immune and breast cancer cells predicts prognosis in breast cancer patients [13]. However, an immune-related gene signature that can accurately predict breast cancer survival outcomes and other clinical features would be greatly beneficial.

**Figure 1:**
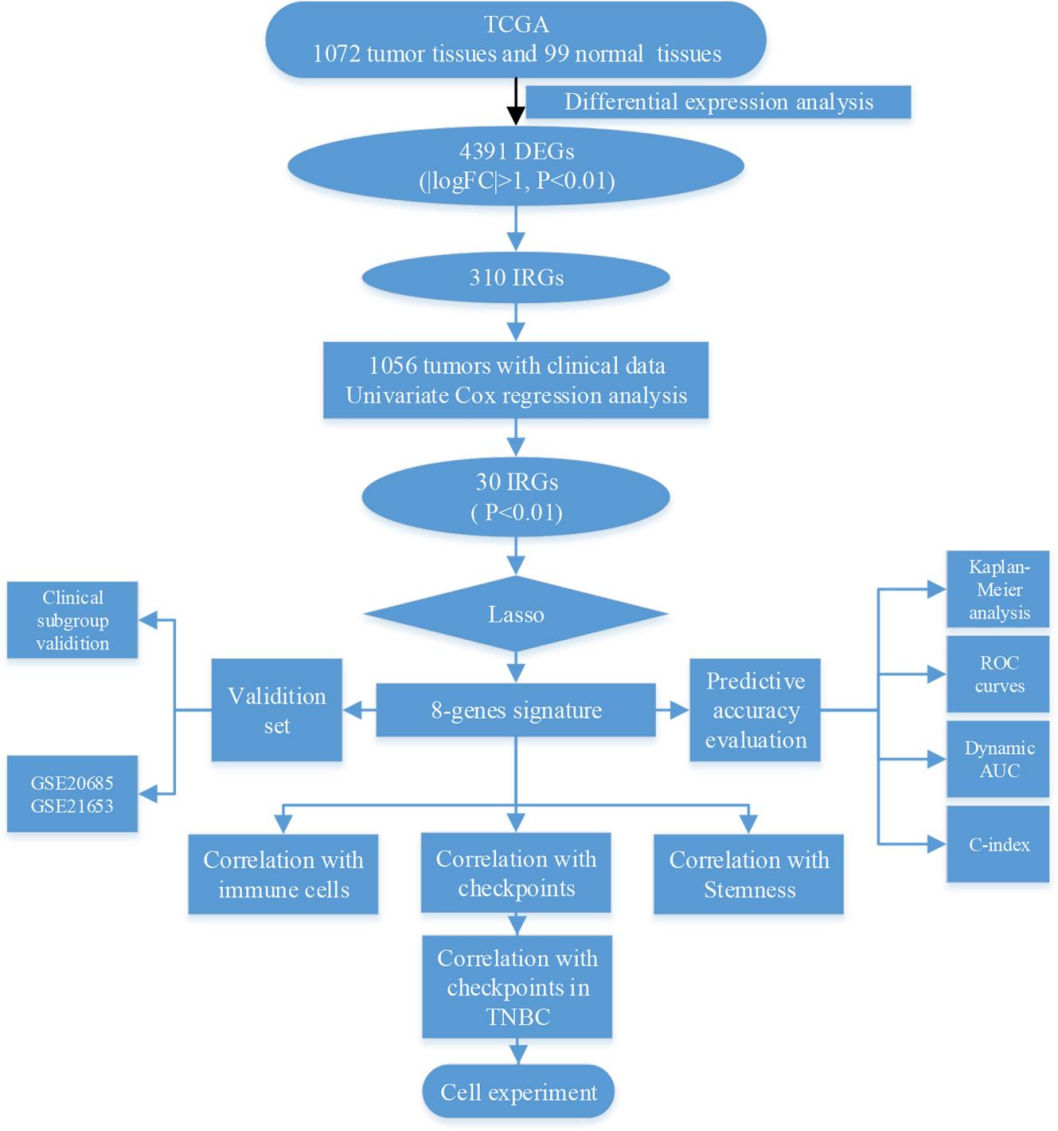
**Schematic of research strategy.**

In this study, we explored whether immune-related genes influence clinical outcomes in breast cancer via immune-related mechanisms (Figure 1). First, we identified 4391 differentially expressed genes (DEGs), of which 310 were immune-related (IRGs), in 1072 breast tumor and 99 normal breast tissues from the TCGA database. Univariate Cox regression analysis of clinical data obtained from 1056 breast cancer patients revealed that the 301 IRGs were statistically significant predictors of survival. Eight of the 301 IRGs were incorporated into a model capable of predicting breast cancer survival outcomes based on Lasso regression analysis. This eight-gene signature was validated in two other breast cancer datasets, and its ability to predict immune checkpoint expression and immune cell infiltration was confirmed using breast cancer cell lines *in* *vitro*.

## RESULTS

### DEGs and IRGs in breast cancer

**Figure 2:**
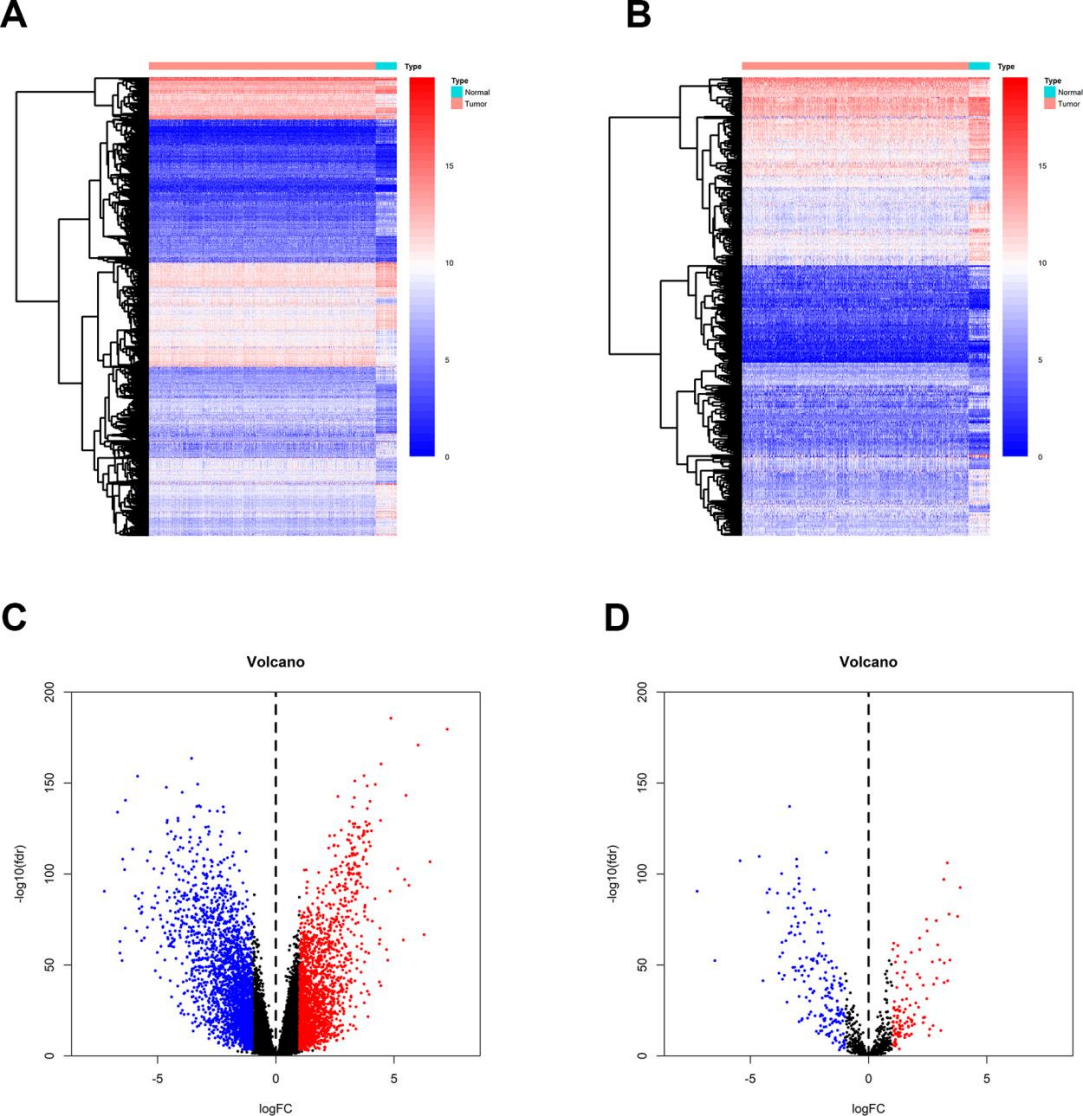
**Identification of DEGs and IRGs between breast tumor and normal breast tissues from the TCGA database.** (**A**, **C**) Heatmap and volcano plot of DEGs between breast tumor and normal breast tissues. (**B**, **D**) Heat map and volcano plot of IRGs between breast tumor and normal breast tissues.

Gene expression data were downloaded from TCGA, and a total of 4391 differentially expressed genes (DEGs) were identified between breast tumor and normal breast tissues (Figure 2A). Out of these 4391 DEGs, 2042 DEGs were over-expressed and 2349 were under-expressed in breast tumor tissues compared to normal tissues (Figure 2C). Using the ImmPort gene list, 310 of the DEGs were identified as immune-related genes IRGs (Figure 2B); of these, 195 genes were under-expressed and 115 were over-expressed in breast tumor tissues compared to normal tissues.

### GO and KEGG enrichment analyses

**Figure 3:**
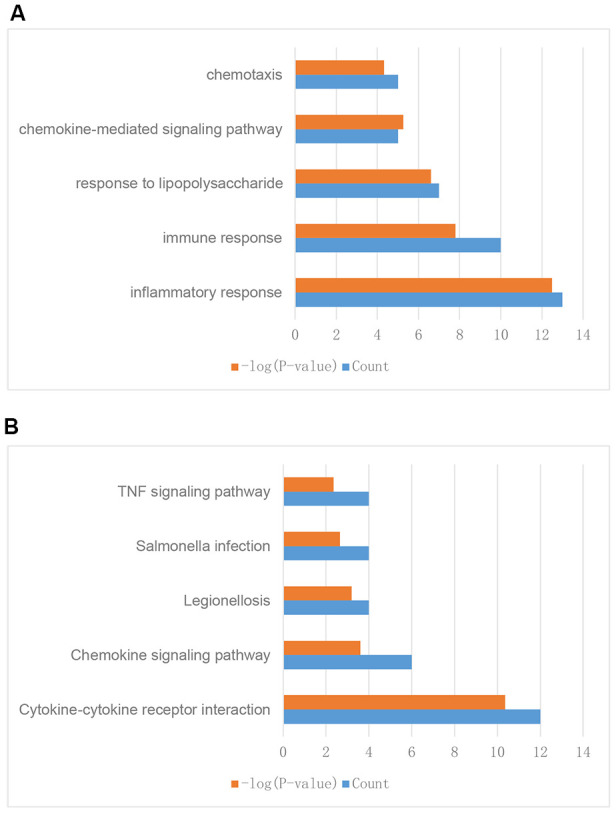
**Enrichment analysis of differentially expressed IRGs between breast tumor and normal breast tissues.** (**A**) GO enrichment analysis results. (**B**) KEGG enrichment analysis results.

To further explore their functions, the 310 IRGs that were differentially expressed between tumor and normal tissues were subjected to GO and KEGG enrichment analyses. GO enrichment analysis indicated that the IRGs were enriched in the following five GO terms: inflammatory response, immune response, response to lipopolysaccharide, chemokine-mediated signaling pathway, and chemotaxis (Figure 3A). These GO terms are associated with immune functions, confirming that these DEGs are immune-related.

KEGG enrichment analysis indicated that the IRGs were enriched in the following five KEGG terms: cytokine-cytokine receptor interaction, chemokine signaling pathway, legionellosis, salmonella infection, and TNF signaling pathway (Figure 3B). These results suggested that these genes might have functions in cell interaction, infection, and other immune related pathways and further validated the GO enrichment analysis results.

### Construction of the eight-IRG signature

**Figure 4:**
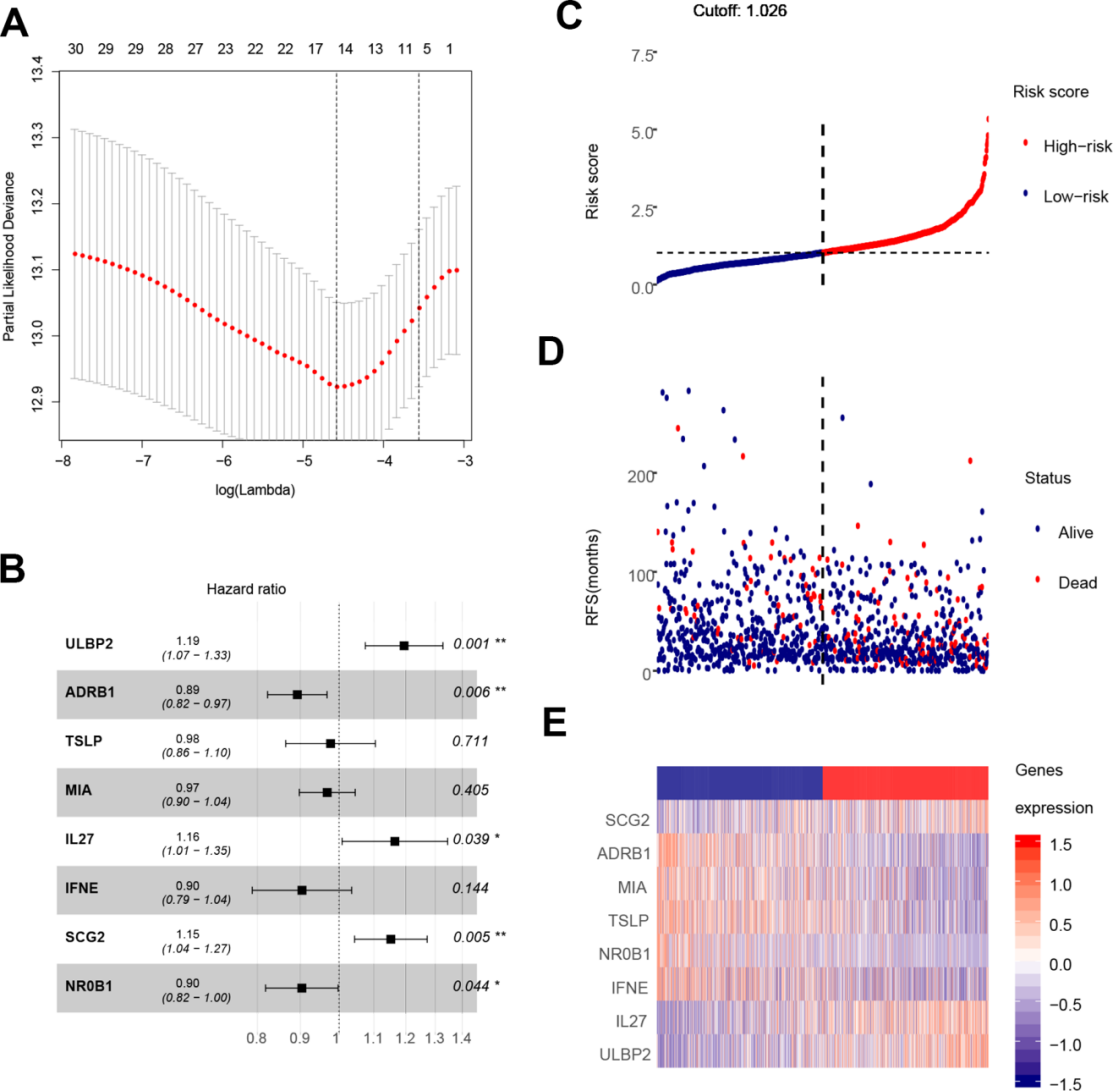
**Construction of the eight-gene signature and performance analysis.** (**A**) Construction of the eight-gene signature. (**B**) Hazard ratio of each gene in the eight-gene signature. (**C**) Risk score distribution and cut-off point. (**D**) Distribution of breast cancer patient survival outcomes. (**E**) Heat map showing expression levels of the eight genes in breast cancer patients.

The analysis process is depicted in Figure 1. As shown in the flow chart, the 310 IRGs were subjected to single factor Cox regression analysis. After considering the statistical significance of associations with OS, five IRGs were selected for further consideration. IRGs that were involved in breast cancer pathogenesis and progression were identified only among the 30 IRGs that were significantly associated with clinical outcomes (Table 1). Finally, using LASSO regression analysis, an eight-IRG signature was constructed in which risk score was calculated using the following formula (Figure 4A):

**Table 1:** General characteristics of breast cancer-specific immune-related genes.

**Gene**	**logFC**	**FDR**	**HR**	**z-value**	**p-value**
ULBP2	1·334234	1·44E-15	1·213258	3·787657	0·000152
ADRB1	-2·43947	6·59E-25	0·865577	-3·58362	0·000339
TSLP	-3·85142	3·11E-90	0·856824	-3·29241	0·000993
MIA	-2·20894	3·00E-14	0·901339	-3·13967	0·001691
IL27	1·777017	1·51E-37	1·213428	2·945851	0·003221
IFNE	-1·07249	7·13E-13	0·826754	-2·86661	0·004149
IL33	-3·65911	3·67E-63	0·90004	-2·77615	0·005501
ULBP1	2·464382	3·77E-32	1·118432	2·665098	0·007697
RXFP1	1·42523	2·38E-17	1·162835	2·630139	0·008535
SCG2	2·309767	1·41E-39	1·13489	2·616983	0·008871
SDC1	2·064759	1·23E-45	1·220534	2·586434	0·009697
IL17B	-2·70568	1·21E-47	0·87877	-2·57143	0·010128
CXCL1	-1·71462	1·07E-12	0·907549	-2·57002	0·010169
VGF	3·344907	5·84E-42	1·093142	2·52823	0·011464
CXCL6	-1·64242	3·68E-13	0·901633	-2·49364	0·012644
NR0B1	-1·87465	5·36E-20	0·88259	-2·48448	0·012974
CXCL2	-4·28926	2·02E-90	0·90192	-2·46882	0·013556
LIFR	-2·94302	2·24E-98	0·852014	-2·46877	0·013558
JUN	-1·55224	5·11E-40	0·826904	-2·42568	0·01528
LGR6	-2·50792	1·59E-23	0·916671	-2·42018	0·015513
TACR1	-3·49715	5·23E-64	0·903367	-2·40346	0·016241
CXCL14	-1·97421	1·45E-14	0·924163	-2·37137	0·017722
NGFR	-2·72487	1·26E-41	0·900896	-2·33917	0·019327
BMP5	-3·63872	7·19E-43	0·923655	-2·25647	0·024041
CXCL3	-2·30754	2·21E-36	0·900084	-2·20137	0·02771
C3	-1·39465	1·49E-20	0·888334	-2·16518	0·030374
SEMA3G	-2·91638	3·15E-91	0·873563	-2·07498	0·037988
TNFRSF8	-1·24776	6·80E-23	0·86757	-2·07424	0·038057
EDN3	-4·4653	4·90E-42	0·944592	-2·05427	0·039949
CCL23	-1·59865	1·44E-25	0·890331	-2·04987	0·040377

$$\begin{array}{l} \text{risk score}=(0.1775\times \text{ULBP}2)-(0.1140\\ \;\;\times \;\text{ADRB}1)–(0.0231\times \text{TSLP})–(0.0324\\ \;\;\times \;\text{MIA})+(0.1522\times \text{IL}27)–(0.1013\times \text{IFNE})\\ \;\;+\;(0.1413\times \text{SCG}2)–(0.1017\times \text{NR}0\text{B}1) \end{array}$$

**Figure 5:**
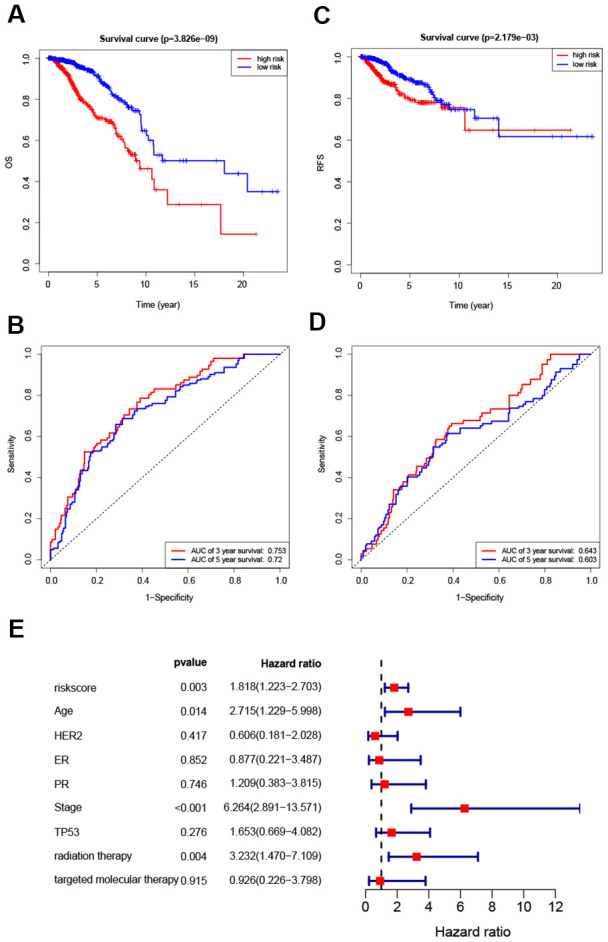
**Survival analysis of high- and low-risk breast cancer patients.** (**A**, **B**) Analysis of OS in high- and low-risk breast cancer patients. (**C**, **D**) Analysis of RFS in high- and low-risk breast cancer patients. (**E**) Hazard ratio of the eight-gene signature and important clinical variables.

Hazard ratios and expression levels for each of the eight genes as well as risk score distributions are shown in Figure 4B–4E. Breast cancer patients were divided into high-risk and low-risk groups using the median risk score as a cut-off point. OS and RFS were shorter in high-risk patients than in low-risk patients (p<0.001) (Figure 5A and 4C). Time-dependent ROC curves indicated that the AUC for three-year and five-year OS were 0.753 and 0.720, while the AUC for three-year and five-year RFS were 0.643 and 0.603 (Figure 5B, 5D). Incorporation of the important clinical variables age, HER2/ER/PR status, stage, TP53 mutation status, therapy type, and risk score into a multivariate regression analysis revealed that risk score was an independent prognostic factor (p=0.003).

### Validation of the eight-gene signature

To further validate the predictive power of our model, we re-evaluated its prediction accuracy in two additional data sets from GEO, GSE20685 and GSE21653. KM curves and survival information revealed significant differences in survival outcomes between high-risk and low-risk patients, confirming the robustness of the eight-IRG signature (Supplementary Figure 1A, 1B).

### Evaluating predictive accuracy of survival outcomes in breast cancer patients

To further explore the predictive capacity of the eight-gene signature, we used it to predict survival outcomes of in different breast cancer patient subgroups (Supplementary Figure 1). Mutations in oncogenes and tumor suppressor genes contribute to malignant behavior in cancer cells [14], and TP53 mutations are very common in breast cancer [15]. Our results revealed a significant difference in survival between high-risk and low-risk patients regardless of TP53 mutation status (Supplementary Figure 1C, 1D). Survival analysis of the breast cancer patients with different disease stages indicated that survival outcomes were significantly worse for both stage I-II and stage III-IV high-risk breast cancer patients than for low-risk breast cancer patients (Supplementary Figure 1E, 1F). This demonstrated that the eight-gene signature could accurately predict survival outcomes in patients with different stages of breast cancer.

Next, we performed survival analysis for breast cancer patients of different pathological subtypes: ER positive or negative, PR positive or negative, and HER2 positive or negative (Supplementary Figure 1E–1L). Survival outcomes were significantly worse for high-risk patients than for low-risk patients regardless of ER and PR status as well as in HER2-negative patients, indicating that the eight-gene signature accurately predicted survival for these pathological types. In addition, although the p value for HER2-positive patients was greater than 0.05, there was an obvious trend towards poorer survival outcomes in high-risk patients compared to low-risk patients of this subtype.

### Associations between eight-gene signature and adjuvant therapies

**Figure 6:**
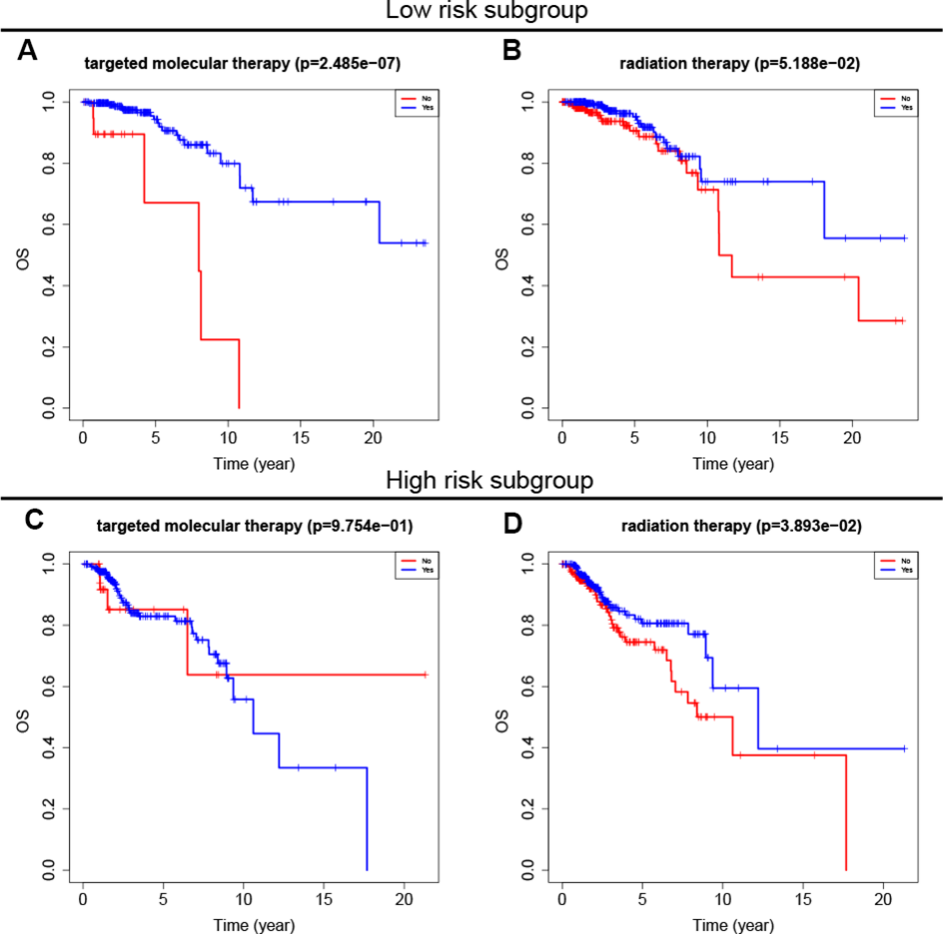
**Survival analysis of adjuvant therapy in high- and low-risk patients.** (**A**, **B**) Survival analysis of targeted molecular therapy and radiation in low-risk patients. (**C**, **D**) Survival analysis of targeted molecular therapy and radiation in high-risk patients.

Adjuvant radiotherapy is often an important component of breast cancer treatment [16]. In addition, radiotherapy can not only reduce the risk of breast cancer recurrence, but also improve prognosis [17]. Targeted molecular therapy has also improved the prognosis of early and advanced stage breast cancer patients over the past 15 years [18]. To explore the relationship between the eight-gene signature and these two treatments, we conducted a subgroup analysis of low-risk and high-risk patients based on treatment type. The results showed that targeted molecular therapy had therapeutic benefits only in low-risk patients, while radiotherapy had therapeutic benefits only in high-risk patients (Figure 6).

### Associations between eight-gene signature and degree of cancer stemness

**Figure 7:**
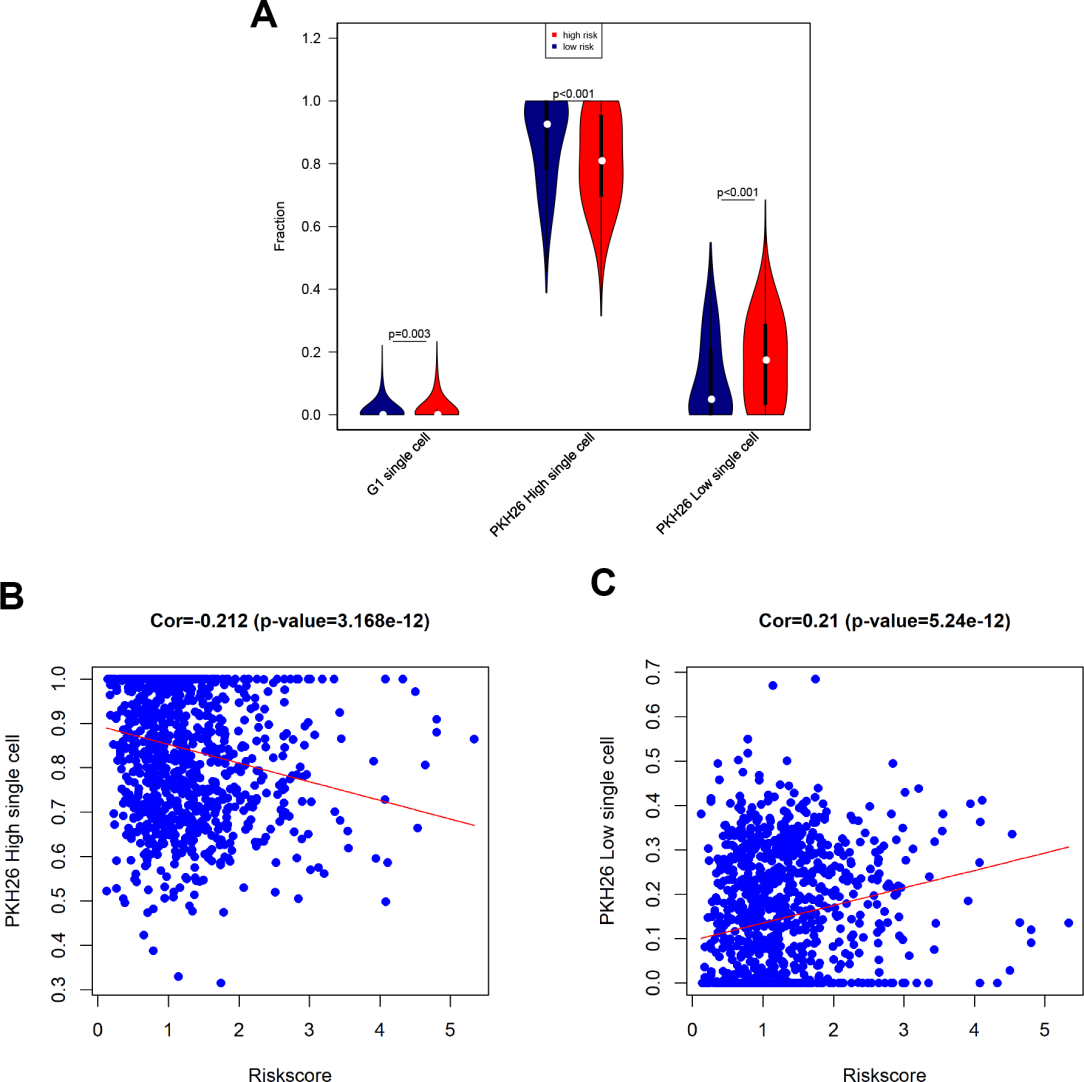
**Associations between eight-gene signature and cancer stemness.** (**A**) Infiltration of G1 phase, high PKH26, and low PKH26 single cells between high- and low-risk patients. (**B**) Analysis of associations between risk score and high PKH26 single cells. (**C**) Analysis of associations between risk score and low PKH26 single cells.

Next, we tested associations between the eight-gene signature and levels of G1 phase, high PKH26, and low PKH26 cells in breast cancer patients using single cell sequencing data [19]. An increase in the population of cells in the G1 cell cycle phase indicates increased proliferation of cancer cells. PKH26 is a biomarker of cancer cell proliferation; cell growth rates and cancer sameness are higher in cancer cells with lower PKH26 expression [19]. Our results revealed that G1 phase cell numbers were slightly increased, low PKH26 cell numbers were increased, and high PKH26 cell numbers were decreased in high-risk breast cancer patients (Figure 7A). Furthermore, risk score was positively correlated with low PKH26 cell numbers and negatively correlated with high PKH26 cell numbers (Figure 7B, 7C). These associations between the eight-gene signature and cancer cell stemness are consistent with the ability of the risk score to predict survival outcomes.

### Associations between eight-gene signature and immune characteristics

**Figure 8:**
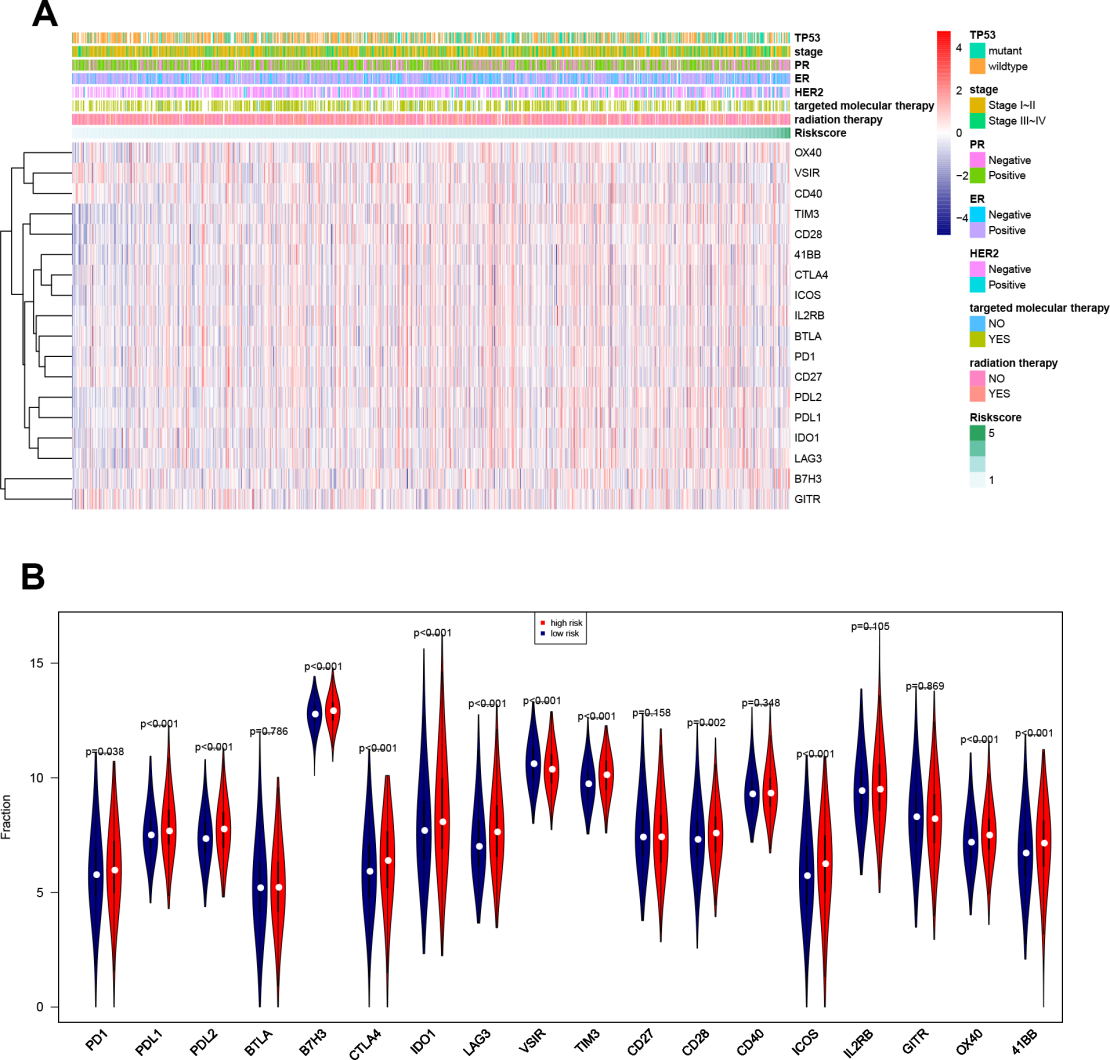
**Associations between eight-gene signature and 18 immune checkpoints.** (**A**) Heatmap showing associations between risk score, clinical variables, and 18 immune checkpoints. (**B**) Predictive value of the eight-gene signature for PD1, PDL2, PDL1, B7H3, CTLA4, IDO1, LAG3, VSIR, TIM3, CD28, ICOS, OX40, and X41BB.

**Figure 9:**
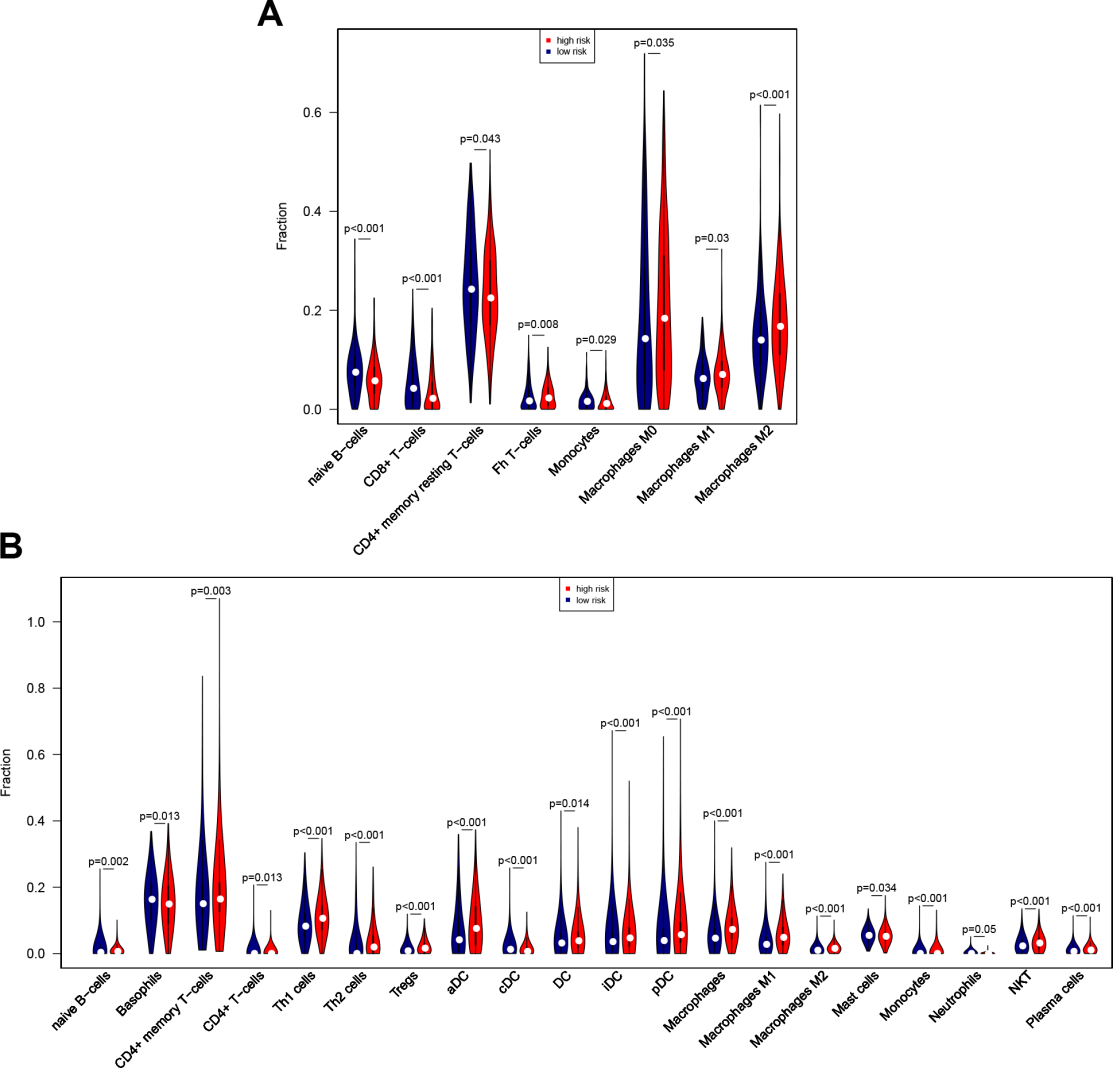
**Associations between eight-gene signature and immune cell infiltration.** (**A**) Predictive value of the eight-gene signature for 8 immune cells based on the CIBERSORT algorithm. (**B**) Predictive value of the eight-gene signature for 21 immune cells based on the xCell algorithm.

Associations between risk score and breast cancer immune characteristics, such as immune checkpoints and immune cell infiltration, were examined (Figures 8, 9). Expression levels were significantly different for 13 of the 18 immune checkpoints tested between high-risk and low-risk breast cancer patients (Figure 8). Among these 13 immune checkpoints, PD1, PDL2, PDL1, B7H3, CTLA4, IDO1, LAG3, TIM3, CD28, ICOS, OX40, and X41BB were expressed at higher levels, while VSIR expression was lower, in high-risk patients compared to low-risk patients.

The CIBERSORT algorithm was then used to identify eight immune cells for which infiltration differed between high-risk and low-risk patients (Figure 9A). The results revealed that naïve B cells, CD8 T cells, resting CD4 memory T cells, and monocytes showed less infiltration, while T follicular helper cells and M0, M1, and M2 macrophages showed more infiltration, in the high-risk group. In an analysis using the xCell algorithm, which uses more detailed immune cell classifications compared to CIBERSORT, a total of 21 immune cells showed significant differences in infiltration between low- and high-risk patients (Figure 9B). Five immune cells with the same definition were identified based on two algorithms, including naïve B cells, monocytes, and M0, M1, and M2 macrophages. The infiltration differences were consistent between the algorithms for four of these five immune cells; although infiltration changes for monocytes were inconsistent between the algorithms, these immune cells show very low infiltration levels overall. In general, the eight-gene signature is therefore predictive of changes in immune cell infiltration.

### Predictive value of the eight-gene signature in TNBC

Additional analyses of both survival outcomes and immune checkpoints were performed in the TNBC subgroup. The results indicated that high-risk non-TNBC patients had poorer survival outcomes than low-risk non-TNBC patients; although the p-value for this comparison was slightly greater than 0.05 in TNBC patients, this trend would likely have reached statistical significance in a larger group of TNBC patients (Supplementary Figure 2A, 2B). Risk score also predicted expression of several immune checkpoints, including PD1, PDL1, PDL2, TIM3, CD28, ICOS, IL2RB, and 41BB, in TNBC patients, and its predictive value in these patients was similar to that observed in the overall breast cancer patient cohort (Supplementary Figure 2C, 2D).

### Validation of eight-gene signature *in* *vitro*

**Figure 10:**
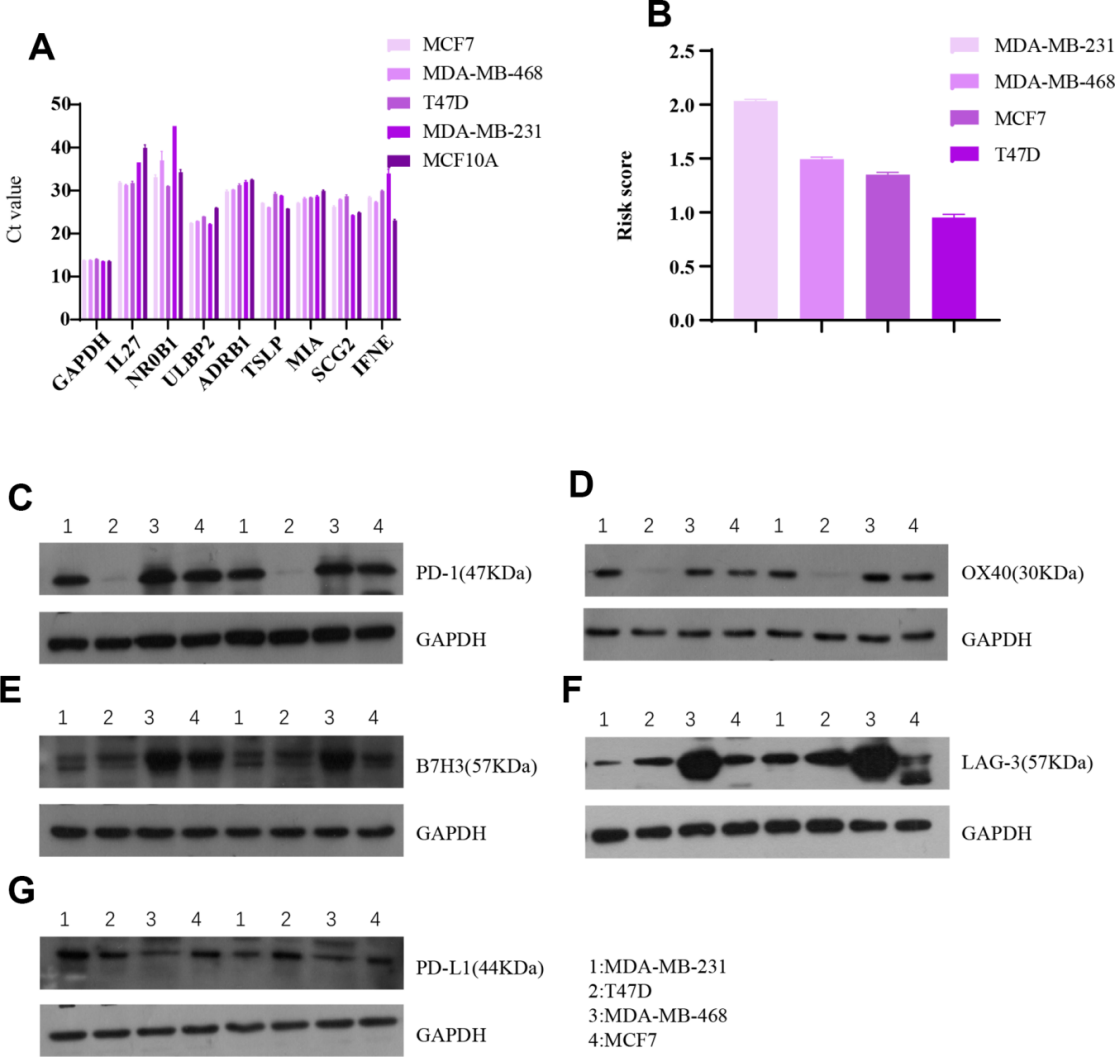
***Invitro* breast cancer cell line experiments validate the predictive value of the eight-gene signature for immune checkpoints.** (**A**) Raw data (Ct value) from Rt-PCR analysis of the eight genes in four breast cancer cell lines and one normal breast cell line. (**B**) Risk scores of the four breast cell lines calculated based on Rt-PCR results. Western blot results for (**C**) PD1, (**D**) OX40, (**E**) B7H3, (**F**) LAG-3, and (**G**) PDL1 expression in the four breast cancer cell lines.

Associations between the eight-gene immune signature and immune checkpoint levels were examined in four breast cancer cell lines (Figure 10). The results indicated that expression of the five immune checkpoints PD1, PDL1, B7H3, LAG-3, and OX40 tended to be lower in cells with higher risk scores. This agrees with our finding that higher risk scores predict higher expression of four immune checkpoints in breast cancer patients.

## DISCUSSION

In this study, we constructed an eight-gene IRG signature that predicted both survival and immune characteristics in breast cancer patients. Enrichment analysis confirmed that these eight genes were involved in immune responses, suggesting that they function by interacting with immune checkpoints or immune cells. Additionally, the gene signature was validated using datasets containing different types of breast cancers, indicating that it may be broadly applicable for many breast cancer patients.

Previous studies have demonstrated that some of the eight genes comprising our signature play important roles in cancer pathology and clinical assessment. ULBP2, a ligand of NKG2D, is associated with poor prognosis in a number of human cancers, and surface expression of this protein is often lost in many human cancer cell types during NK cell-mediated cytolysis [20–22]. The TSLP signaling pathway interacts with other immune pathways and may promote survival of breast and pancreatic cancer cells, although its effects in breast cancer remain poorly understood [23–25]. The MIA gene family is considered a useful marker for many types of cancers, and its upregulation has been associated with shorter progression free survival times [26, 27]. Here, we found that upregulation of TSLP and MIA was associated with better survival outcomes, which contradicts previous studies indicating that these genes interact with other pathways to promote proliferation and growth of breast cancer cells. IL27 has been identified as a potentially useful target for anti-cancer clinical applications, probably due to its ability to regulate CD8+ T cells, natural killer cells, macrophages, and other immune checkpoints [28, 29]; our present findings regarding IL27 are consistent with these prior studies. NR0B1 sensitizes lung cancer cells to chemotherapy and inhibits their invasive abilities [30, 31]; similar effects might explain the poorer survival outcomes observed here in breast cancer patients upon downregulation of this gene.

Although some of the genes included in our signature may not play important roles in clinical cancer pathology and assessment, they do play roles in other diseases. ADRB1 is implicated in cognitive neural diseases, possibly due to its role in neuroinflammatory processes, and is also associated with heart failure [32–34]. These effects might also have contributed to the survival outcomes seen here. IFNE is a member of the interferon family that inhibits proliferation in various cells by regulating NK cells and the JAK-STAT pathway [35]. Upregulation of this gene in the present study might therefore contribute to improved survival outcomes. SCG2 may be a biomarker of bipolar disease and is known to regulate hypertension in humans [36], perhaps explaining the poorer survival outcomes observed here following upregulation of this gene.

In this study, we also identified pathways through which the eight IRGs might affect breast cancer outcomes. Several proteins from the TNF signaling pathway play important roles in breast cancer and its treatment [37]. For example, TNF-α is implicated in immune responses in breast cancer and might therefore serve as a treatment target in triple negative breast cancer [38, 39]. Moreover, chemokine signaling pathway members, especially CCL5, are involved in the pathogenesis and development of breast cancer [40–42]. Although not all of the genes included in the enrichment analysis were incorporated into the eight-IRG signature, they were differentially expressed in breast tumor and normal tissues. Because the signature was constructed from these genes, the pathways in which they are enriched are relevant to the eight-IRG signature and may represent important differences between the two tissue types. These pathways might therefore reveal biological processes responsible for the immune functions of these genes as well as potential mechanisms that contribute to survival outcomes in breast cancer patients.

The eight-gene signature was capable of predicting both a number of immune checkpoints which may serve as biomarkers and the infiltration of immune cells that can act as therapeutic targets in breast cancer. Previous studies strongly support the use of PD1 and PDL1 as targets for breast cancer treatment [43, 44]. Overexpression of CTLA4 can increase numbers of Treg cells and thereby influence breast cancer pathogenesis and development [45]. In addition, tumor-associated macrophages are associated with poor prognosis in breast cancer patients [46], which is consistent with our present finding that increased infiltration of M0, M1, and M2 macrophages was associated with poorer survival outcomes. Evidence also suggests that CD4 and CD8 T cells can act as biomarkers and therapeutic targets for breast cancer treatment [47, 48], which is in keeping with our finding that higher risk scores based on the eight-IRG signature were associated with higher levels of CD8 and resting CD4 memory T cells. Since these immune checkpoints and cells can serve as targets for immunotherapy [49], the ability of our eight-IRG signature to predict these immune characteristics might prove valuable in the clinical setting.

Programmed death receptor 1 (PD1), which is mainly expressed in activated T lymphocytes and myeloid cells, and its ligand PD-L1 are important immunosuppressive molecules [50, 51]. The binding of PD1 to its ligand can inactivate T cells, leading to immune escape reactions in tumors [50]. Single or combined drug therapies using immune checkpoint inhibitors (ICIs) play anti-tumor roles by blocking the transmission of immunosuppressive signals, reactivating the immune response of T cells to tumors, and restoring immune activity in the tumor microenvironment [52]. The advent of immunotherapy has changed treatment regimens for many tumors, including breast cancer, and clinical trials of TNBC inhibitors have yielded encouraging results. Higher risk scores based on our eight-IRG model tended to be associated with poorer survival outcomes in TNBC group, indicating that this gene signature might help predict prognosis in TNBC patients.

In conclusion, we developed an eight-gene signature using IRGs that were differentially expressed between breast tumor and normal breast tissues. This signature predicted breast cancer survival outcomes for various pathological types at different clinical stages. The genes included in the signature were also associated with immune checkpoint expression and immune cell infiltration. Our eight-gene signature therefore accurately predicted both immune characteristics and survival outcomes in breast cancer patients.

## MATERIALS AND METHODS

### Accessing gene expression data from TCGA

TCGA is a cancer gene expression database accessible to all cancer researchers and clinicians. We downloaded clinical data for 1056 breast cancer patients and mRNA level gene expression data for 1072 breast tumor tissues and 99 normal breast tissues from the TCGA database. Clinical data was reordered and is summarized in Table 2. Because TCGA is open access and contains publicly available, ethical approval is not required before use.

**Table 2:** Clinical characteristics of the patients in TCGA data sets.

**Characteristics**	**Number(%)**
Age	
<=60	586(55·5)
>60	470(44·5)
HER2	
Positive	108(14·7)
Negative	625(85·3)
ER	
Positive	777(77·0)
Negative	232(23·0)
PR	
Positive	676(67·2)
Negative	330(32·8)
Stage	
I~II	773(74·7)
III~IV	262(25·3)
TP53 status	
Wildtype	513(67.0)
mutant	253(33.0)
Radiation	
Yes	540(55.6)
No	432(44.4)
Targeted molecular therapy	
Yes	516(91.7)
No	47(8.3)
Survival status	
Survival	907(85·9)
Dead	149(14·1)
Relapse status	
Relapse-free	793(89·6)
Relapse	92(10·4)

### Differential expression analysis and identification of IRGs

ImmPort is a publicly available database accessible to professionals specializing in immunology [53]. Differential expression analysis of RNA-Seq data from the 1072 breast tumor and 99 normal breast tissues was conducted using the R package “limma” from Bioconductor [54]. We applied |logFC|<1 and P<0.01 as the criteria to identify DEGs and determined which DEGs were also IRGs based on the IRG list downloaded from ImmPort.

### Enrichment analysis

GO enrichment analysis was performed to identify biological functions, while KEGG enrichment analysis was used to identify both biological functions and pathways, associated with the DEGs [55, 56]. The R package “ClusterProfiler” was used for both enrichment analyses [57].

### Construction and validation of the IRG signature

Cox regression is a widely used tool for survival analysis [58]. Based on differential expression data, 30 IRGs were identified that contributed to survival outcomes in the 1056 breast cancer patients for which clinical data was available. Least Absolute Shrinkage and Selection Operator (Lasso) regression is a useful method for weighting model parameters and helps identify the most important variables to generate the best predictive model. The “glmnet” R package was used to carry out the LASSO Cox regression analysis [59]. This analysis identified an eight-gene signature that we used to construct a model that predicted both immune characteristics and clinical outcomes in breast cancer patients. Risk scores were calculated for each sample based on coefficients assigned to each prognostic IRG in the signature. The median risk score was used as a cut-off value for dividing training and validation group patients into high- and low-risk groups.

### Performance analysis

The Kaplan-Meier (KM) survival curve is a powerful tool for analyzing patient survival outcomes [60]. In this study, the R package “survival” was used to generate the KM survival curve. A Receiver Operating Characteristic (ROC) curve is often used to evaluate the sensitivity and specificity of a model in predicting outcome events [61]. We used the R package “survival ROC” to conduct ROC analysis. Using the median risk score as a cut-off and plotting clinical outcome data for breast cancer patients against their risk scores, we generated an ROC curve and calculated area under curve (AUC) values for both 3- and 5-year survival. An AUC value between 0·5 and 0·7 indicates evidence of a successful model, values between 0·7 and 0·9 indicate strong evidence of a successful model, and values greater than 0·9 indicate very strong evidence of a successful model.

### Validation using the GEO database

The Gene Expression Omnibus (GEO) database is an open access database containing datasets from published projects [62, 63]. In our study, OS data for 328 patients from the GSE20685 dataset and RFS data for 249 patients from the GSE21653 dataset were used as validation groups [64–66].

### Analysis of cancer stemness using single cell sequencing data

Single cell sequencing is a new method for generating sequencing profiles for specific cell types [67]. GSE124989 includes single cell sequencing data from three breast cancer cell subtypes, enabling analysis of degree of stemness in breast cancer cells [19]. The CIBERSORTX algorithm generates signatures from single cell sequencing data that allow the calculation of numbers of individual cell types from bulk RNA sequencing data [68]. We used the CIBERSORTX algorithm to analyze GSE124989 data and constructed signatures for the following breast cancer cell subtypes: G1 phase, high PKH26, and low PKH26 single cells. We then used the signatures of these three cell types to evaluate cancer stemness in breast cancer patients.

### Evaluation of predictive accuracy among different clinical stages and pathological types of breast cancer

Clinical stage and pathological type are important factors that influence clinical decisions. We therefore tested the ability of the eight-gene signature to predict the survival outcomes in patients with different clinical stages and pathological types of breast cancer. Clinical stages were grouped as stage I-II and stage III-IV, while the pathological types were classified as ER positive or negative, PR positive or negative, and HER2 positive or negative. KM survival analysis was used to analyze clinical outcomes in the different breast cancer patient subgroups.

### Associations between eight-gene signature and immune characteristics

Correlation analysis was then conducted to explore the eight-gene signature’s ability to predict immune checkpoint expression and immune cell infiltration. Breast cancer patients were divided into high- and low-risk groups the based on the cut-off risk score value before analyzing associations with immune characteristics.

We analyzed the correlation between the eight-gene signature and the expression of the 18 immune checkpoints identified as existing or potential targets for cancer immunotherapy. T-tests were used to compare the mean immune checkpoint expression levels between high- and low-risk patients.

The CIBERSORT algorithm is used to estimate the proportion of specific cell types based on bulk gene expression data [68]. LM22 is a leukocyte gene signature comprised of 547 genes that distinguishes 22 human immune cell subsets with high accuracy. The XCell algorithm calculates infiltration of 64 immune cells from based on RNA-seq data [69]. Using these algorithms, we evaluated amounts of immune cells belonging to these 22 and 64 immune cell subsets in each sample at a significance level of p<0.05; only samples that exceeded that threshold were included in our study. Infiltration of the 22 and 64 immune cell subsets was compared between high- and low-risk breast cancer patients using t-tests.

### *In vivo* validation of results

*In vitro* experiments using MCF7, MDA-MB-468, T47D, and MDA-MB-231 breast cancer cell lines as well as the MCF10A normal breast cell line as external reference (all from Genechem, Shanghai) were conducted to further validate the immune-checkpoint prediction accuracy of the eight-gene signature. Real-time quantitative PCR (Rt-PCR) was performed to quantify expression of the eight genes in the signature using GAPDH as an internal reference gene (Table 3). Relative RNA expression levels for genes in the signature were calculated via the 2^-ΔΔCt^ method. Promega M-MLV and Trizol (Pufei, Shanghai) kits were used in this experiment, and primers were obtained from Ribobio (Guangzhou).

**Table 3:** Primer sequences used in qPCR in the cell experiment.

**Genes**	**Upstream primer sequence**	**Downstream primer sequence**	**Amplified fragment size (bp)**
GAPDH	TGACTTCAACAGCGACACCCA	CACCCTGTTGCTGTAGCCAAA	121
ULBP2	CCGCTACCAAGATCCTTCTG	GGATGACGGTGATGTCATAGC	109
ADRB1	TCTCGGCCCTGGTGTCCTT	GCCCGGTTGGTGACGAAGT	115
TSLP	CTAACCTTCAATCCCACCG	CTGAGTTTCCGAATAGCCT	108
MIA	CGAAGTTTGGGACTGGTTTAG	GGCAGACAGCAAGATGATGAC	179
IL27	CGCTTTGCGGAATCTCACC	AGGGCATGGAAGGGCTGAA	158
IFNE	AGCCGATGTCTGTTCTTTGTG	CCTCGGGCTTCTAAACTCTGT	108
SCG2	CTGAAGCAAAGACCCACTG	TGTACTCCAAAGCCCTGAT	180
NR0B1	CCAAGGAGTACGCCTACCTCA	CATTTCCAGCATCATATCATCCA	272

The R package “sva” was used for batch normalization of the Rt-PCR results with TCGA data. Risk scores were then calculated for each breast cancer cell line. Protein expression levels for the immune checkpoints PD1, B7H3, LAG-3, OX40, and PDL1, and the internal reference GAPDH in the breast cancer cell lines were assessed by western blot, and associations with risk score were examined. Antibodies for these proteins were purchased from Abcam (Shanghai).

### Statistical analysis

Statistical analyses were conducted using the R program from the R project for statistical computing (https://www.r-project.org/) and SPSS. The R package “pheatmap” was used to plot heatmaps, and “ggpubr” was used to generate boxplots. Unless otherwise indicated, p<0.05 was used to indicate statistical significance.

## Supplementary Material

Supplementary Figures
