# Supplementary material for: Eight immune-related genes predict survival outcomes and immune characteristics in breast cancer
Source: Aging (Albany NY). 2020 Aug 3;12(16):16491–513. doi: 10.18632/aging.103753 (PMC7485735; doi:10.18632/aging.103753)
Supplement: Supplementary Figures [file aging-12-103753-s001..pdf]

SUPPLEMENTARY FIGURES

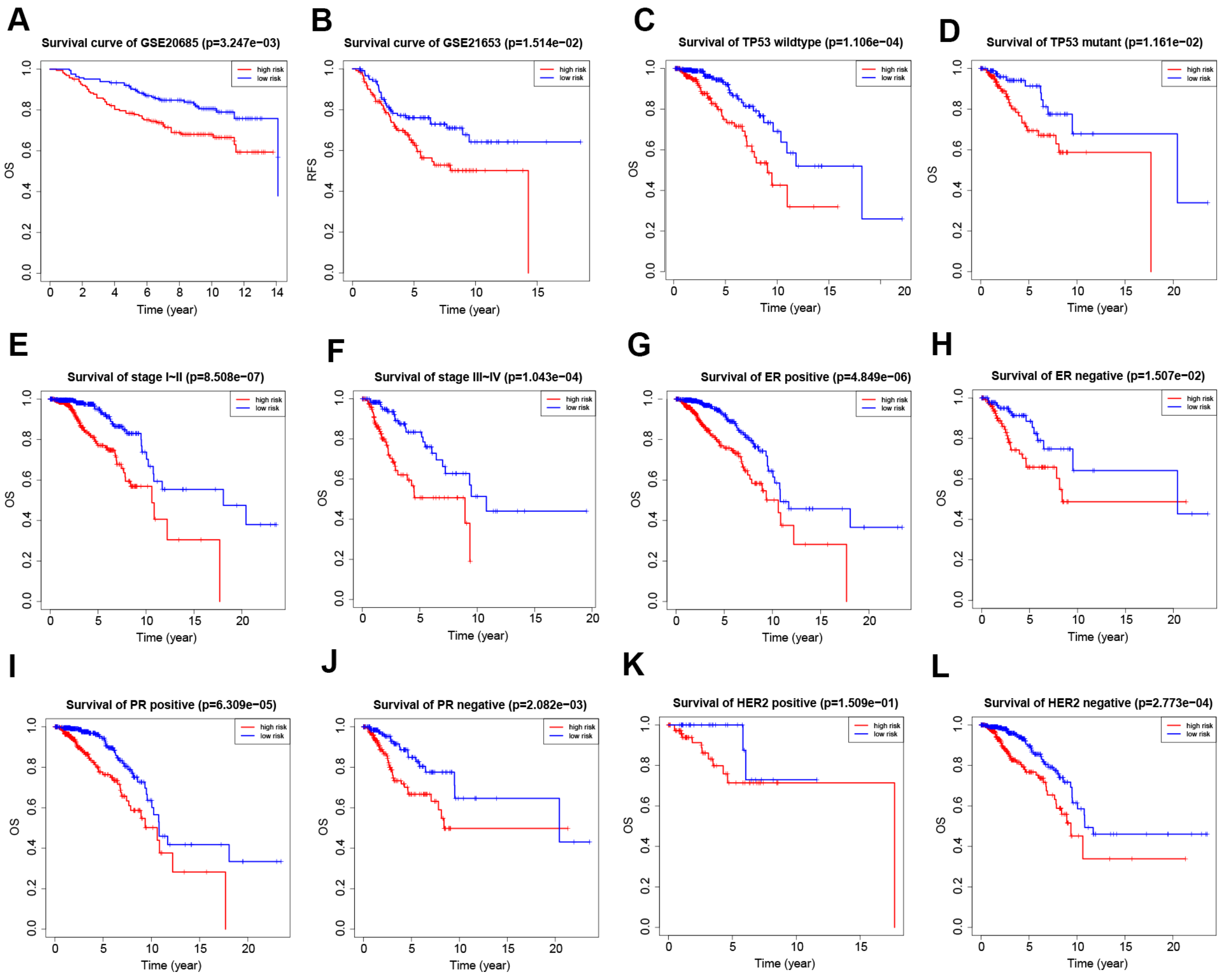

Supplementary Figure 1. Validation of the eight-gene signature using survival analysis of GEO datasets and in different subtypes and stages of breast cancer. (**A**, **B**) Survival analysis of the GSE20685 and GSE21653 GEO datasets. (**C**, **D**) Survival analysis of breast cancer patients depending on TP53 mutation status. (**E**, **F**) Survival analysis of stage I-II *vs*. stage III-IV breast cancer patients. (**G**–**L**) Survival analysis in breast cancer patients with the following subtypes: ER positive/negative, PR positive/negative, HER2 positive/negative.

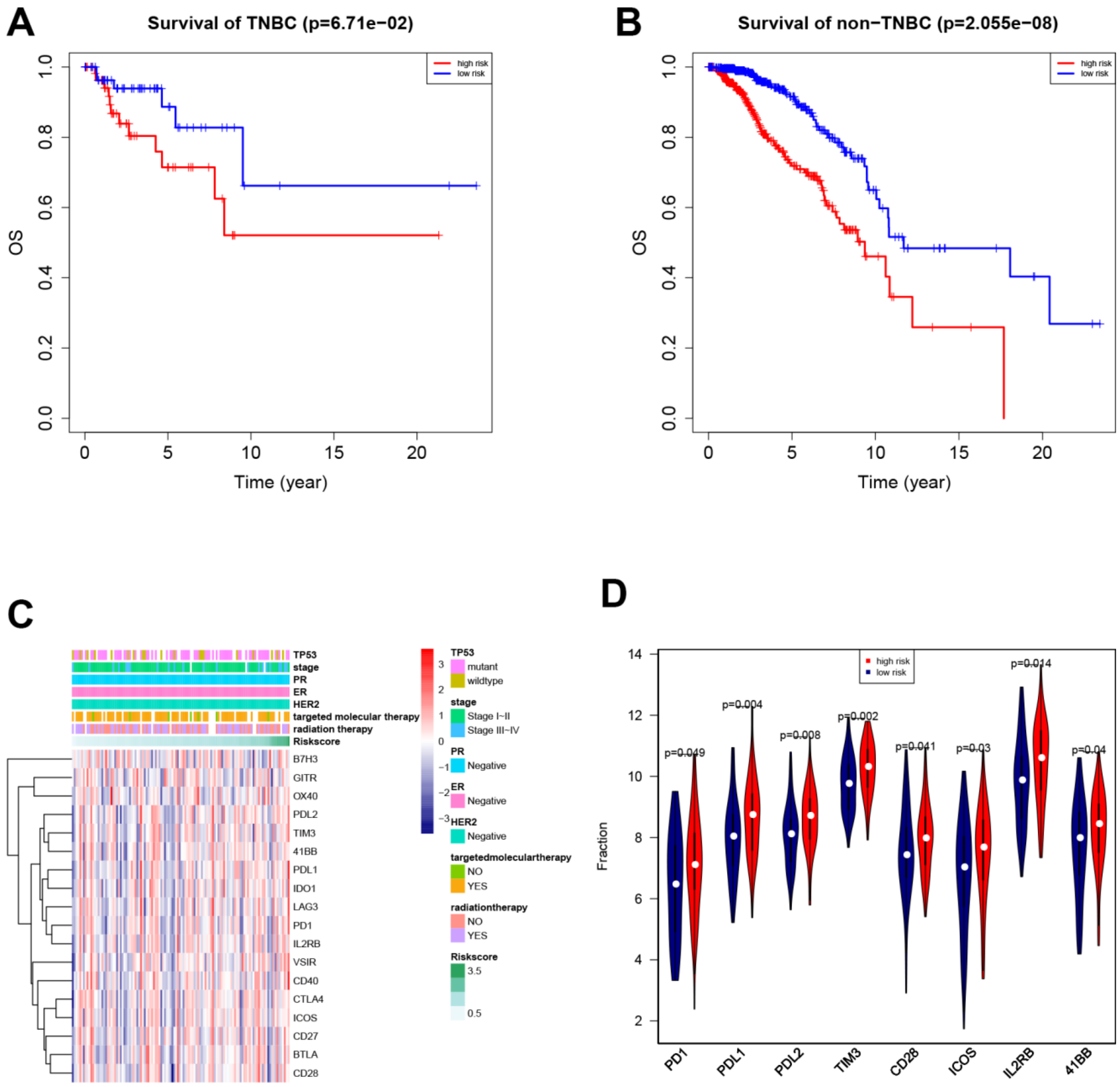

Supplementary Figure 2. Predictive value of the eight-gene signature for survival outcomes and immune checkpoints in TNBC subgroup. (**A**) Survival analysis of high- and low-risk TNBC patients. (**B**) Survival analysis of high- and low-risk non-TNBC patients. (**C**) Heatmap showing associations between risk score, clinical variables, and 18 immune checkpoints. (**D**) Predictive value of the eight-gene signature for PD1, PDL1, PDL2, TIM3, CD28, ICOS, IL2RB, and 41BB.
